# RECOMMENDATIONS REGARDING THE DESIGN OF PAIN ASSESSMENT AND MANAGEMENT TOOL FOR OLDER ADULTS WITH HEART FAILURE

**DOI:** 10.1093/geroni/igad104.1935

**Published:** 2023-12-21

**Authors:** Youjeong Kang, Yong Kyung Choi, Seung-Eun Oh

**Affiliations:** University of Utah, Salt Lake City, Utah, United States; University of Pittsburgh, Pittsburgh, Pennsylvania, United States; Seoul Women’s College of Nursing, Seoul, Republic of Korea

## Abstract

Nearly 80% of all patients with heart failure (HF) are older adults (≥65 years of age) who are at high risk for frequent hospitalizations. Older adults with HF may delay in responses to pain that could trigger the burden of HF symptoms such as dyspnea due to the increased cardiovascular workload and oxygen demands. However, there is a concern that healthcare providers may be poorly prepared to assess and manage pain when reported by specific populations. This qualitative secondary analysis examined the design recommendations of HF providers for a comprehensive home-based pain assessment and management tool for older adults with HF. The study used in-depth interviews with 20 healthcare providers specializing in HF across different states. Participants provided various recommendations, including a checklist of questions about the patient’s past experience with pain, their living environment, and any activities that trigger pain. While some participants preferred electronic tools, such as an mHealth app, for data collection and analysis, they acknowledged the limitations of using technology for older adults. The tool should be straightforward, easy to navigate, and involve patients in the design process to ensure their motivation and buy-in. The tool should also consider the caregiver’s input. In conclusion, this study highlights the importance of a comprehensive assessment of pain symptoms and daily activities to provide personalized care for older adults with HF. The design recommendations provided by healthcare providers can aid in the development of a unique home-based pain assessment and management tool for older adults with HF.

